# Intranasal insulin enhances brain functional connectivity mediating the relationship between adiposity and subjective feeling of hunger

**DOI:** 10.1038/s41598-017-01907-w

**Published:** 2017-05-09

**Authors:** Stephanie Kullmann, Martin Heni, Ralf Veit, Klaus Scheffler, Jürgen Machann, Hans-Ulrich Häring, Andreas Fritsche, Hubert Preissl

**Affiliations:** 1 Institute for Diabetes Research and Metabolic Diseases of the Helmholtz Center Munich at the University of Tübingen, Tübingen, Germany; 2 German Center for Diabetes Research (DZD e.V.), Tübingen, Germany; 3 Institute of Medical Psychology and Behavioral Neurobiology, University of Tübingen, Tübingen, Germany; 4 Department of Internal Medicine IV, University of Tübingen, Tübingen, Germany; 5 Department of High-Field Magnetic Resonance, Max Planck Institute for Biological Cybernetics, Tübingen, Germany; 6 Department of Biomedical Magnetic Resonance, University of Tübingen, Tübingen, Germany; 7 Institute for Diabetes and Obesity, Helmholtz Diabetes Center at Helmholtz Zentrum München, German Research Center for Environmental Health (GmbH), Neuherberg, Germany; 8 Institute of Pharmaceutical Sciences, Department of Pharmacy and Biochemistry, Eberhard Karls Universität Tübingen, Tübingen, Germany

## Abstract

Brain insulin sensitivity is an important link between metabolism and cognitive dysfunction. Intranasal insulin is a promising tool to investigate central insulin action in humans. We evaluated the acute effects of 160 U intranasal insulin on resting-state brain functional connectivity in healthy young adults. Twenty-five lean and twenty-two overweight and obese participants underwent functional magnetic resonance imaging, on two separate days, before and after intranasal insulin or placebo application. Insulin compared to placebo administration resulted in increased functional connectivity between the prefrontal regions of the default-mode network and the hippocampus as well as the hypothalamus. The change in hippocampal functional connectivity significantly correlated with visceral adipose tissue and the change in subjective feeling of hunger after intranasal insulin. Mediation analysis revealed that the intranasal insulin induced hippocampal functional connectivity increase served as a mediator, suppressing the relationship between visceral adipose tissue and hunger. The insulin-induced hypothalamic functional connectivity change showed a significant interaction with peripheral insulin sensitivity. Only participants with high peripheral insulin sensitivity showed a boost in hypothalamic functional connectivity. Hence, brain insulin action may regulate eating behavior and facilitate weight loss by modifying brain functional connectivity within and between cognitive and homeostatic brain regions.

## Introduction

Interactions between the central and peripheral nervous system influence various metabolic and cognitive functions^1, 2^. In this process, numerous hormonal signals play an important role to exert their effects on eating behavior and body weight^2^. Insulin is a major peripheral hormone released after food intake and the reduced ability of insulin to act on its target tissue, i.e. insulin resistance (IR), is a hallmark of type 2 diabetes mellitus (T2D) and obesity. Moreover, there is growing evidence that IR is an important link between metabolic diseases and deteriorating cognitive function^3–5^. While there is considerable evidence on the pathomechanisms of peripheral IR, central or brain IR is much less characterized in humans. It is clear meanwhile that insulin action in the brain goes beyond the homeostatic center (i.e. the hypothalamus) extending to other brain regions. To unravel the role of brain insulin action in humans, the intranasal approach is an efficient tool to deliver the peptide directly to the CNS compartment, practically bypassing the body periphery^6^. Neuroimaging revealed that intranasal insulin administration modulates brain networks involved in homeostatic control, reward processing, and cognitive control functions, thus, influencing different aspects of human behavior (for review see refs 4, 7, 8). Furthermore, intranasal insulin produces multiple behavior and metabolic effects^9^. Acutely applied intranasal insulin decreases energy intake^10^ and wanting for sweet foods^11^. It intensifies satiety^12^, modulates olfactory and gustatory sensitivity^13, 14^ and even improves peripheral insulin sensitivity^15^. This is in line with animal studies confirming the central catabolic action of insulin (e.g. refs 16–18). Studies on cognitive effects indicate that hippocampal-dependent memory processes benefit particularly from intranasal insulin in humans^19–24^. Similarly, rodent studies have shown that insulin plays an important role in regulating synaptic plasticity in the hippocampus^25, 26^. More recently, evidence accumulates that it is possible to improve cognitive functions in patients with T2D and early Alzheimer’s disease by enhancing brain insulin action using intranasal insulin^20–23, 27–31^. Furthermore, the observed improvement in cognitive functions in T2D patients is related to an increase in functional connectivity within the default-mode network (DMN), which is linked to higher cognitive functions as decision-making and memory^31^. The DMN comprises a brain network exhibiting high functional connectivity between the frontal, lateral parietal cortices, precuneus and the temporal cortex including the hippocampus. Numerous studies have pointed to disruptions in functional connectivity within the DMN especially in neurological and neuropsychiatric disorders^32^. However, also T2D and obesity are related to altered functional connectivity of the DMN preceding brain atrophy and cognitive impairment^33–36^. Furthermore, evidence has accumulated that, in cognitively healthy young overweight and obese humans, insulin resistance affects regions of the DMN^4^. Besides the hypothalamus, obesity-associated brain IR affects predominantly higher cognitive brain regions especially the prefrontal regions included in the DMN^11, 37–39^. Furthermore, a recent study has shown that the central hubs of the DMN, the posterior cingulate cortex and medial prefrontal cortex, are functionally closely connected to the hypothalamus^40, 41^. However, it is currently not clear whether it is possible to boost functional connectivity of the DMN in young overweight and obese brain insulin resistant individuals to improve their eating behavior and metabolism.

We hypothesize that intranasal insulin may acutely enhance signaling in the DMN and between the hypothalamus and the DMN in healthy young lean, overweight and obese adults. Furthermore, we hypothesize that this modification is related to peripheral metabolism and eating behavior. For this purpose, we acquired resting-state functional magnetic resonance imaging (fMRI) to measure functional connectivity of the brain in response to intranasal insulin and placebo administration.

## Results

We evaluated insulin versus placebo-induced changes in functional connectivity in the DMN in lean, overweight and obese participants 30 min after spray application. For this purpose, we used four different DMN seed regions for the analyses (Supplementary Figure 1). This included the core regions and the prefrontal regions of the DMN, as the prefrontal cortex (PFC) is particular insulin responsive. The functional connectivity (FC) map of each network is shown in Supplementary Figure 2. We additionally included a peripheral insulin sensitivity index, assessed by an oral glucose tolerance test on a separate day, as a covariate into the design. Hence, we were able to evaluate potential interactions between central and peripheral insulin sensitivity.

**Figure 1:**
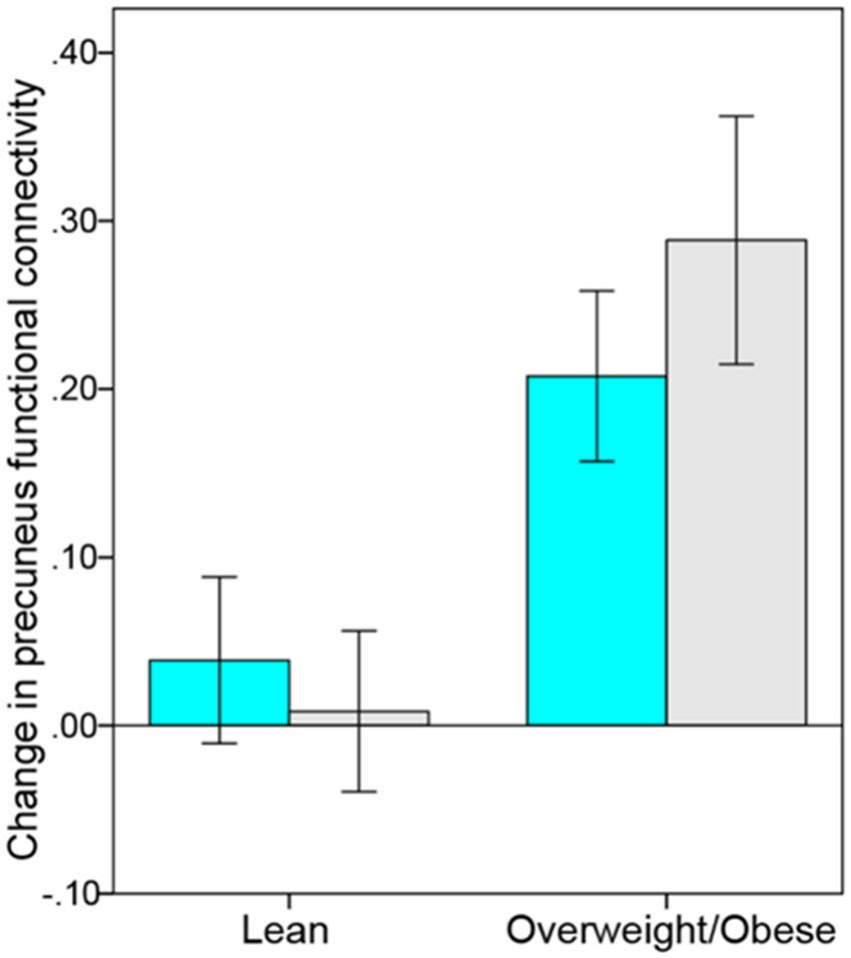
Change in functional connectivity in default-mode network (DMN) based on the posterior cingulate seed region after intranasal insulin (cyan) and placebo (gray) spray. Overweight and obese participants showed a stronger increase in precuneus functional connectivity (rs*fMRI2* minus rs*fMRI1*) on both insulin and placebo day compared to lean participants (p_FWE_ < 0.05, whole-brain corrected).

We observed a main effect of group (body weight) in the precuneus within the default-mode network (F = 18.30; p_FWE_ < 0.05 whole-brain corrected; MNI coordinates x: 0 y: −54 z: 60) (Fig. 1). Overweight/obese compared to lean participants revealed increased FC between the posterior cingulate cortex and the precuneus on both insulin and placebo day.

### Insulin-induced changes in functional connectivity in the default-mode network

**Figure 2:**
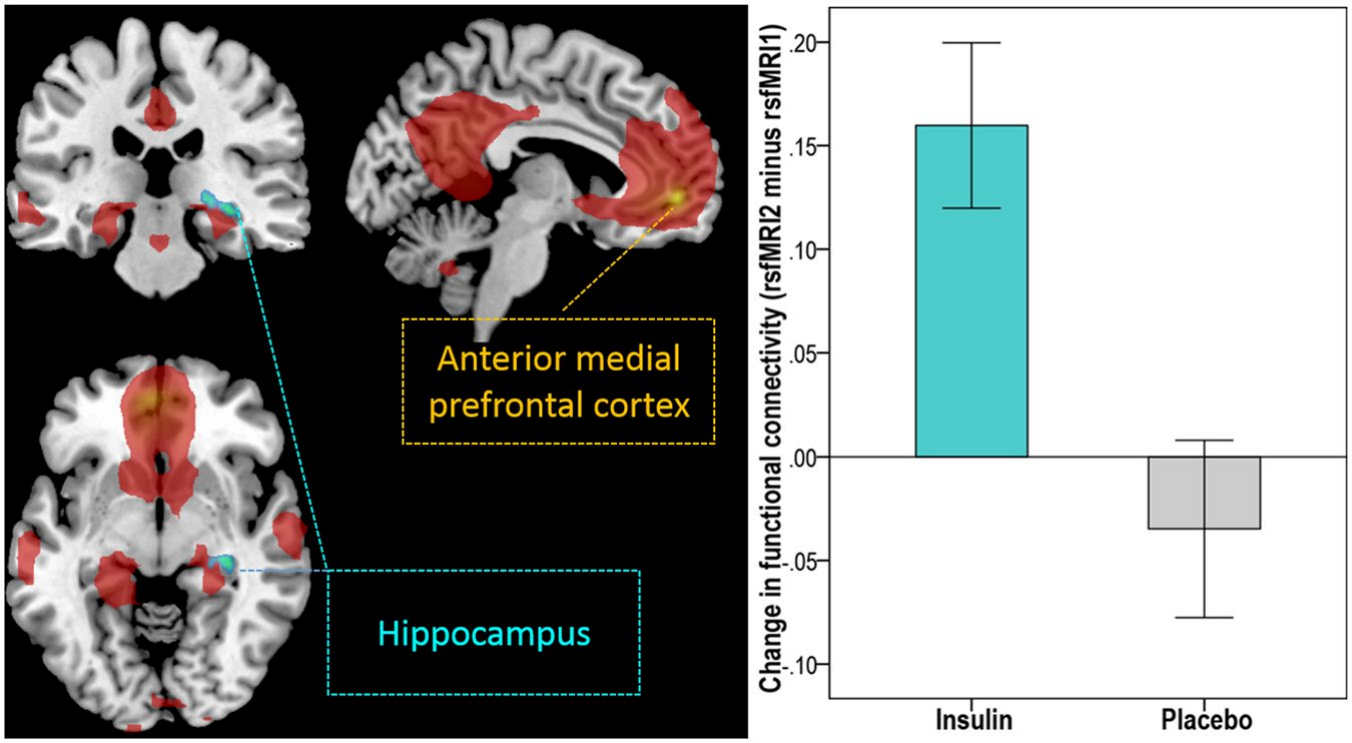
Intranasal insulin increased functional connectivity between the anterior medial prefrontal cortex (PFC) of the default-mode network (DMN) and the hippocampus in lean, overweight and obese participants. Group-averaged DMN of lean and overweight/obese participants under baseline condition (rs*fMRI1*) for the anterior medial PFC seed region is displayed in red (p < 0.05, FWE whole-brain corrected). Cyan-color coded region reveals voxels within the right hippocampus showing a significant change in functional connectivity within the DMN based on the anterior medial PFC seed region after intranasal insulin compared to placebo (p_FWE_ < 0.05 small volume corrected). Bar plot on right shows the change in functional connectivity after intranasal insulin and placebo (rs*fMRI2* minus rs*fMRI1*) between the anterior medial PFC and the hippocampus in lean, overweight and obese participants (i.e. the extracted differential correlation coefficients of the right hippocampus).

**Figure 3:**
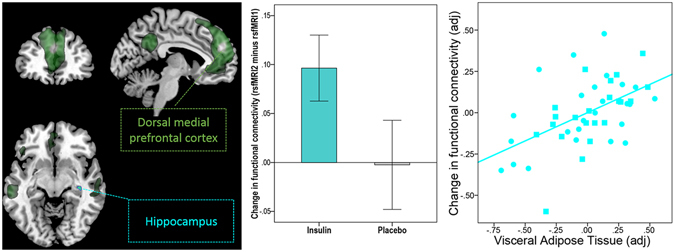
Intranasal insulin increases functional connectivity between the dorsal medial prefrontal cortex (PFC) of the default-mode network (DMN) and the hippocampus in lean, overweight and obese participants. Group-averaged DMN of lean and overweight/obese participants under baseline condition (rs*fMRI1*) for the dorsal medial PFC seed region is displayed in dark green (p_FWE_ < 0.05, whole-brain corrected). Cyan-color coded region reveals voxels within the right hippocampus showing a significant change in functional connectivity within the DMN based on the dorsal medial PFC seed region after intranasal insulin compared to placebo (p_FWE_ < 0.05 small volume corrected). Bar plot in the middle shows change in functional connectivity after intranasal insulin and placebo (rs*fMRI2* minus rs*fMRI1*) between the dorsal medial PFC and the hippocampus in lean, overweight and obese participants (i.e. the extracted differential correlation coefficients of the right hippocampus). Partial correlation plot on the right shows significant correlation between visceral adipose tissue and the intranasal insulin induced change in functional connectivity between dorsal medial PFC and hippocampus adjusted for total intracranial volume, sex, age and BMI (p < 0.001, r_adj_ = 0.529). Circles represent lean participants and squares represent overweight and obese participants.

We observed a significant main effect of condition (insulin vs placebo) within the default-mode network, resulting in increased functional connectivity after intranasal insulin compared to placebo application in the hippocampus (p_FWE_ < 0.05 small volume corrected). Specifically, insulin increased functional connectivity between the anterior medial PFC and the right hippocampus (F = 13.85; p_FWE_ < 0.05 small volume corrected; MNI coordinates x: 33 y: −27 z: −9) (Fig. 2) and between the dorsal medial PFC and the right hippocampus (F = 15.44; p_FWE_ < 0.05 small volume corrected; MNI coordinates x: 33 y: −21 z: −9) (Fig. 3). No significant group differences (body weight) were observed. Furthermore, we performed multiple regression analyses to investigate the relationship between the change in FC with hunger rating scores and different fat compartments. The change in FC between the dorsal medial PFC and the hippocampus significantly correlated with visceral adipose tissue (VAT) (T = 3.9, p < 0.001; r = 0.496; r_adj_ = 0.529 adjusted for BMI, age, sex, and total intracranial volume (TICV)) and with the change in subjective feeling of hunger 120 minutes after intranasal insulin spray application (T = −3.6, p = 0.001; r = −0.476; r_adj_ = −0.504 adjusted for BMI, age, sex, and TICV). No such correlations were observed for change in FC between the anterior medial PFC and the hippocampus and on the placebo day.

**Figure 4:**
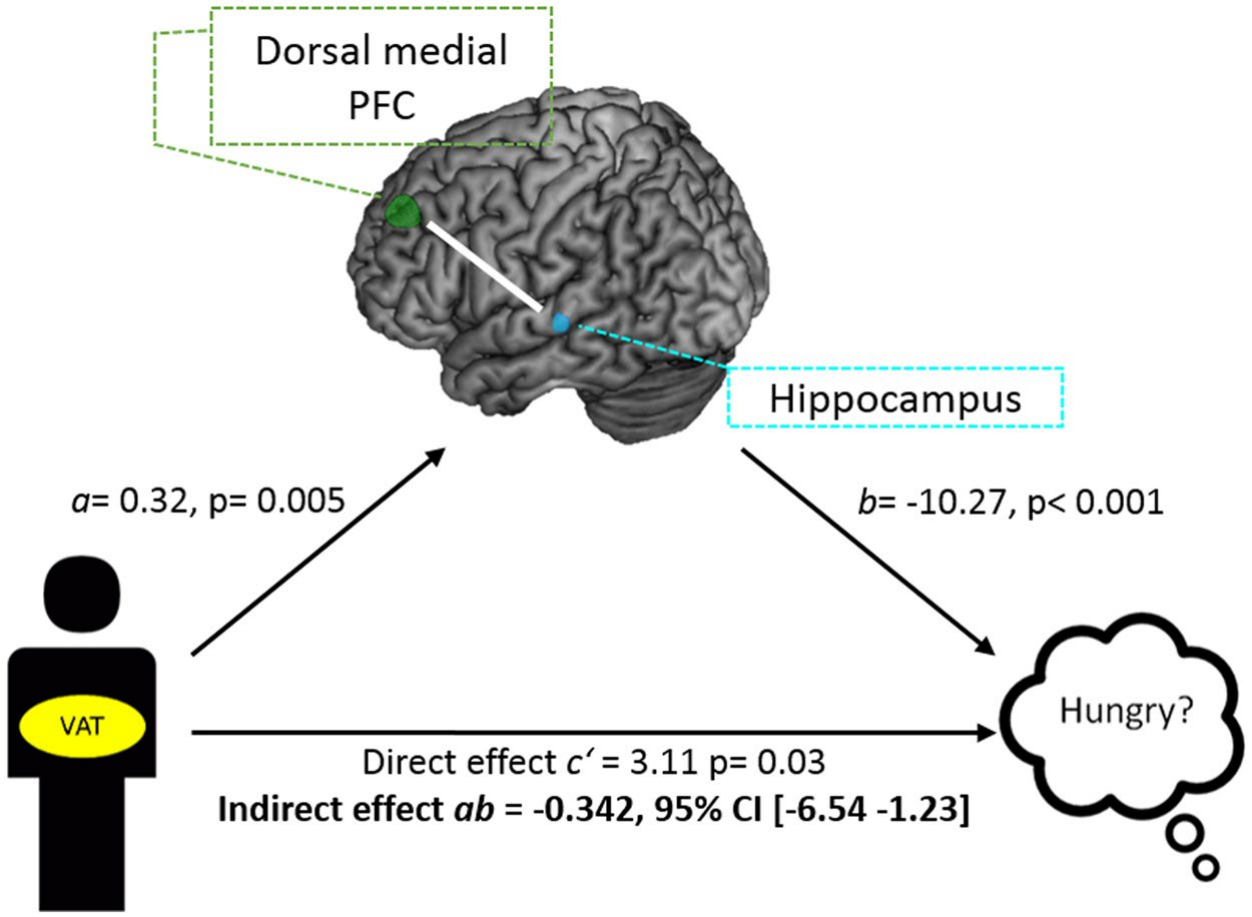
Model of visceral adipose tissue (VAT) as a predictor of subjective feeling of hunger mediated by functional connectivity change after insulin spray application. There is a significant indirect effect of VAT on the change of hunger via the change in functional connectivity between the dorsal medial prefrontal cortex and hippocampus 120 min after insulin. Path coefficients and corresponding p-values are shown next to arrows; path a indicates the relationship between VAT and insulin-induced hippocampus functional connectivity change, path b indicates the relationship between the insulin-induced hippocampus functional connectivity change and change in hunger; path ab indicates the indirect effect of VAT on hunger via the insulin-induced hippocampus functional connectivity change; path c’ indicate the direct effect of VAT on the change in hunger after insulin.

Mediation analyses was performed to test whether the increase in FC between the dorsal medial PFC and the hippocampus induced by intranasal insulin served as a mediator between abdominal fat (i.e. VAT) and hunger. This analysis revealed a significant negative indirect effect of VAT on hunger 120 min after intranasal insulin application via the dorsal medial PFC-hippocampus functional connectivity change (standardized indirect effect *ab* = −0.34, 95% Bootstrap CI −6.54 to −1.23; Fig. 4). In other words, the increase in functional connectivity induced by intranasal insulin suppressed the relationship between VAT and hunger. No such relationship was observed on the placebo day.

### Insulin-induced changes in functional connectivity between the default-mode network and the hypothalamus

**Figure 5:**
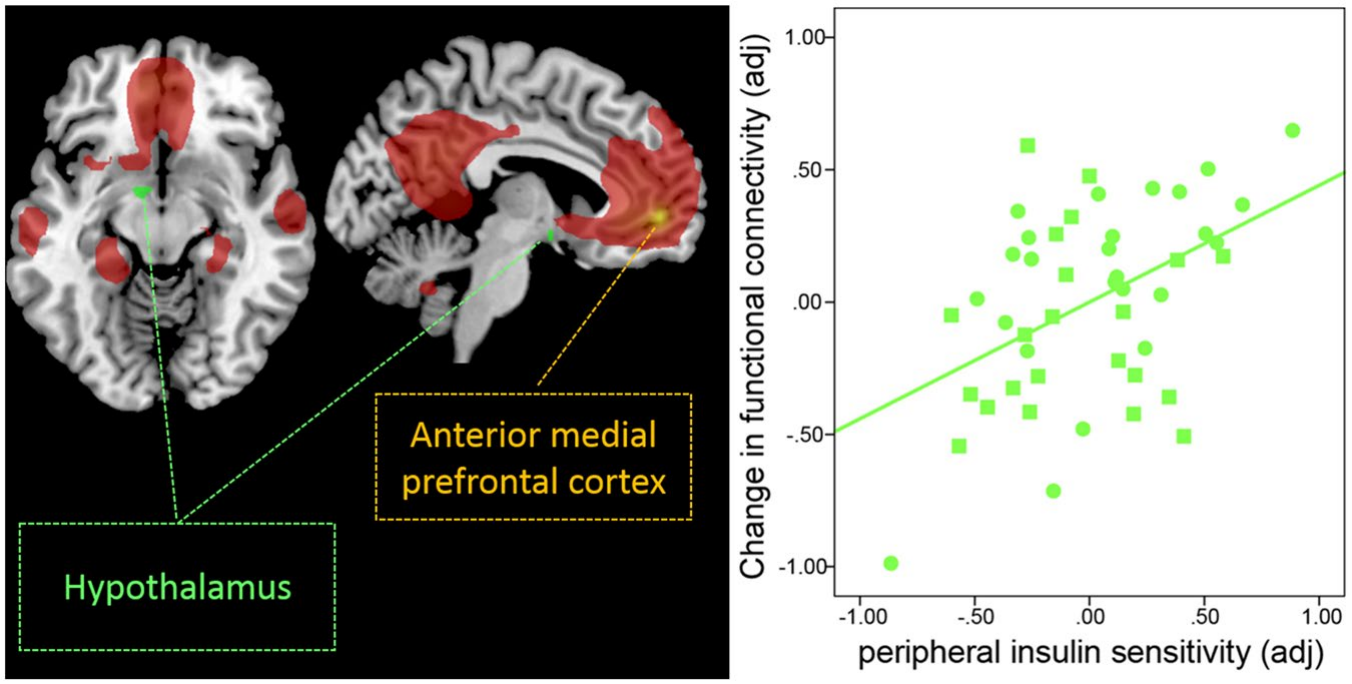
Intranasal insulin increases functional connectivity (FC) between the hypothalamus and the anterior medial prefrontal region of the default-mode network in peripherally insulin sensitive participants. Group-averaged DMN of lean and overweight/obese participants under baseline condition (rs*fMRI1*) for the anterior medial prefrontal cortex (PFC) seed region is displayed in red (p_FWE_ < 0.05, whole-brain corrected). Bright green color-coded region reveals voxels within the hypothalamus showing a significant change in functional connectivity with the anterior medial PFC of the DMN, as identified by a significant interaction between condition (insulin versus placebo spray) and the peripheral insulin sensitivity index (p_FWE_ < 0.05 small volume corrected). Partial correlation plot on the right shows a significant positive correlation between peripheral insulin sensitivity index and the change in functional connectivity between the anterior medial PFC and hypothalamus. With higher peripheral insulin sensitivity, we observed a stronger increase in functional connectivity adjusted for total intracranial volume, sex, age and visceral adipose tissue (r_adj_ = 0.459, p = 0.002). Circles represent lean participants and squares represent overweight and obese participants. No such relationship was observed after placebo administration.

We observed a significant interaction between condition (insulin versus placebo) and peripheral insulin sensitivity in the hypothalamus (Fig. 5). Insulin increased functional connectivity after intranasal application compared to placebo between the anterior medial PFC and the hypothalamus (F = 10.84; p_FWE_ < 0.05 small volume corrected; MNI coordinates x: −6 y: 0 z: −9) only in participants with high peripheral insulin sensitivity. The change in hypothalamic FC after insulin spray application significantly correlated positively with peripheral insulin sensitivity (T = 3.3, p = 0.002; r = 0.476; r_adj_ = 0.459, adjusted for VAT, age, sex, and TICV) (Fig. 5). No such relationship was observed on placebo day.

## Discussion

In healthy young lean, but also in overweight and obese participants, intranasal insulin acutely enhanced functional connectivity between the prefrontal regions of the DMN and the hippocampus. These regions are known to be crucial for higher cognitive processes. Interestingly, we found increased dorsal medial PFC-hippocampal functional connectivity to significantly modulate hunger. This insulin-induced increase acted as a mediator between individual’s visceral adipose tissue, a metabolic unfavorable abdominal fat depot, and subjective feeling of hunger. Participants with a stronger increase in functional connectivity felt less hungry 120 minutes after insulin administration. Furthermore, individuals with more visceral adipose tissue were hungrier. This relationship was suppressed by the increase in functional connectivity between the dorsal medial PFC and hippocampus. Moreover, the connection between metabolic and cognitive centers of the brain could be boosted by intranasal insulin, however only in participants with high peripheral insulin sensitivity. The higher the peripheral insulin sensitivity index, the stronger the increase in functional connectivity between the anterior medial prefrontal cortex of the DMN and the hypothalamus after insulin administration.

Neuroimaging studies investigating target brain regions of insulin action, using endogenous and exogenous stimulation of insulin, have identified the hypothalamus, striatal and frontal regions to be particularly insulin sensitive^4, 7^. Specifically, in response to glucose ingestion, individuals with a higher increase in endogenous insulin showed a more pronounced prefrontal cortex activity decrease to food cues^37, 42, 43^. Similarly, the PFC and hypothalamus neural activity attenuated in response to intranasal insulin^11, 15, 39^. Moreover, not just localized neural activation but also functional connectivity of the hypothalamus and PFC can be modulated by glucose^43, 44^ or by meal ingestion^45–47^. In the fasting compared to the fed state^46^ hypothalamic-PFC functional connectivity was enhanced, while the ingestion of a meal reduced functional connectivity to the medial and lateral PFC^45, 47^. Interestingly, other hormonal interventions with leptin^48, 49^ or intranasal oxytocin^50–52^ have likewise shown to alter PFC functional connectivity. Leptin replacement therapy in patients with lipodystrophy, for example, significantly increased hypothalamic-PFC functional connectivity accompanied by normalization of eating behavior^49^. Accordingly, in the current study we found the functional connection between the anterior medial PFC and the hypothalamus to increase in participants with favorable peripheral insulin sensitivity. Hence, evidence accumulates that brain-periphery interactions play an important role in the regulation of eating behavior and metabolism. With respect to insulin action, recent studies have shown that the prefrontal and hypothalamic response to insulin stimulation are significantly correlated with peripheral insulin sensitivity^11, 15, 37, 39, 42^. Moreover, intranasal insulin delivery to the brain improved peripheral insulin sensitivity in placebo-controlled hyperinsulinemic-euglycemic glucose clamps^15, 53^. This coincides with the current finding that central insulin administration can enhance the functional connectivity between cognitive and homeostatic brain centers in peripherally insulin sensitive individuals.

The insulin-induced heightened functional connectivity between the prefrontal regions of the DMN and the hippocampus was independent of peripheral IR. Lean, overweight and obese participants with poor and high peripheral insulin sensitivity showed an increase in functional connectivity after intranasal insulin administration. Consistent with this notion, Fadel and Reagan^25^ proposed that hippocampal IR may occur independent of peripheral IR. Rats with hippocampal-specific IR showed no changes in body weight and peripheral insulin sensitivity, however changes in neural plasticity and impaired spatial learning^54^. Interestingly, in the current study, we observed a link between central insulin action in the hippocampus and metabolism and hunger. The increased hippocampal functional connectivity acted as a mediator between perceived hunger and visceral adipose tissue. This is in line with animal and humans studies showing that the hippocampus processes visceral energy-status relevant information detecting interoceptive signals of hunger and satiety^55, 56^. Hippocampal neurons form memory of a meal and are involved in inhibitory effects of recent eating on subsequent food consumption^56, 57^. Concomitantly, patients with hippocampal lesions will rate their subjective state of hunger in the middle of the magnitude scale independent of when the last meal was consumed^55, 58^. Moreover, reduced sensitivity to internal signals is found in obesity^59^ and in individuals with high western diet consumption^60^. Specifically participants with self-reported high fat and high refined-sugar diet performed poorer on hippocampal sensitive memory tasks and were less accurate in recalling what they had previously eaten^60^. Free-fatty acids (FFAs), which can be mildly suppressed by intranasal insulin^61^, may mediate the relationship between visceral adipose tissue and brain insulin responsiveness. Specifically, elevated levels of saturated nonesterified fatty acids led to a diminished insulin reactivity in theta frequency activity generated in the hippocampus^62^. Hence, it is possible that circulating FFAs in individuals with high visceral adipose tissue interferes with the subjective feeling of hunger. Enhancing hippocampal functional connectivity by means of centrally acting insulin could potentially breach this viscous cycle improving internal awareness for hunger. Hence, we propose that the beneficial effects of boosting hippocampal functional connectivity with intranasal insulin go beyond improving cognition, as recently reported^31^. It can potentially improve metabolism by enhancing the sensitivity to internal signals, thereby reducing perceived hunger. Hence, intranasal insulin could be a potential therapeutic tool to improve memory formation for food and raise internal awareness for satiety and hunger signals.

While solid evidence exists that obesity-associated IR affects at least parts of the DMN^4^, the current study showed for the first time that DMN functional connectivity plays a pivotal role in central insulin action in cognitively healthy young adults. The prefrontal part of the DMN, in particular, may constitute a link between networks controlling insulin-mediated effects on metabolism and cognition. However, besides brain IR, peripheral IR seems to play an important role in DMN function. Even in cognitively intact prediabetes and T2D patients, the severity of peripheral IR is related to altered brain function in regions of the DMN showing reduced cerebral blood flow and disrupted functional connectivity^63^. Interestingly, treated T2D patients do not show reduced cerebral blood flow in the DMN compared to the insulin resistant controls^64^. Hence, it could be that altered brain functions in regions of the DMN is a consequence of peripheral IR rather than a cause of brain IR. Concurrently, we identified obesity-associated changes in functional connectivity in the posterior part of the DMN, which was not influenced by intranasal insulin. Hence, we propose the anterior part of the DMN to constitute an overlap between peripheral and central IR. However, further studies are necessary to investigate cause and consequences of central and peripheral IR.

A limitation of the study is the missing examinations on cognitive functions. All of our participants were students at the university and displayed no psychiatric or neurological illness. Furthermore, we used a thorough metabolic screening to exclude type 2 diabetes and other alterations associated with the metabolic syndrome. Nonetheless, to further link metabolic and cognitive endpoints of brain insulin action, further studies are necessary evaluating metabolism as well as cognition. Moreover, we cannot rule out a possible spillover of intranasal insulin into the peripheral blood^15, 53, 65^. More frequent blood sampling is needed to detect this phenomenon. We recently characterized this spillover at systemic elevated insulin levels. Even after mimicking the spillover effect during the placebo condition, intranasal insulin still significantly mediated whole-body metabolism^53^. However, no detailed kinetics have yet been reported at fasting insulin levels.

Taken together, we were able to show that acute administration of intranasal insulin administration can enhance DMN functional connectivity in healthy lean, overweight and obese adults. The insulin induced increase in dorsal medial PFC-hippocampal functional connectivity served as a mediator, suppressing the relationship between visceral adipose tissue and hunger. Furthermore, we observed a significant brain-periphery interaction of insulin action. Only individuals with an insulin-induced DMN-hypothalamic functional connectivity change revealed favorable peripheral insulin sensitivity. Therefore, enhancing brain functional connectivity using intranasal insulin has potential to boost cognition and metabolism. The relevance of our findings for the treatment of obesity and metabolic disease is currently however still speculative. Our results point to a novel mechanism of how brain insulin action facilitates weight loss by enhancing brain functional connectivity and reducing perceived hunger. Hence, intranasal insulin could be a potential therapeutic tool to improve internal awareness for satiety and hunger signals. Further intervention studies have to be conducted to demonstrate who will benefit from enhancing brain insulin action.

## Methods

### Participants

The study sample consisted of 25 healthy lean and 10 overweight and 12 obese adult participants (BMI range 19–40 kg/m^2^), who were also part of a recent study investigating brain insulin action using cerebral blood flow^11^. Overweight and obese participants were required to have a BMI greater than 25 kg/m². One obese subject could not be analyzed due to missing functional MRI data. The Ethics Committee of the Medical Faculty at the University of Tübingen approved the protocol and informed written consent was obtained from all participants. All participants were students at the University of Tübingen recruited using broadcast emails. The study was registered as clinical trial (NCT01797601; February 18^th^ 2013). To assure that participants were healthy and did not suffer from psychiatric, neurological nor metabolic diseases, they underwent a thorough medical examination. To assess body fat distribution of the participants, whole-body MRI measurements were obtained at a 1.5-T whole body imager (Magnetom Sonata; Siemens Healthcare, Erlangen, Germany) (for further description please see Kullmann *et al*.^11^). To assess peripheral insulin sensitivity, all participants underwent a 75 g oral glucose tolerance test with blood drawing at five time points after an overnight fast prior to the intranasal experiment. Insulin sensitivity was estimated according to Matsuda and DeFronzo^66^. The study was performed in accordance with the relevant guidelines and regulations. Participants characteristics are summarized in Table 1.

**Table 1:** Participants’ characteristics.

	Lean group	Overweight/Obese group	p
Gender (female/male)	10/15	11/11	—
Age (y)	25.88 ± 3.30	26.81 ± 3.62	0.360
Body mass index (kg/m²)	22.59 ± 1.99	30.57 ± 3.51	<0.001
oGTT-derived insulin sensitivity index (AU)	16.0 ± 7.6	10.66 ± 6.56	0.005
HbA1c (% and mmol/mol)	5.2 ± 0.3/33.15 ± 3.1	5.3 ± 0.3/33.9 ± 2.7	0.380
Whole-body MRI (in liter)			
Total adipose tissue	17.82 ± 4.41	40.5 ± 10.7	<0.001
Visceral adipose tissue	1.55 ± 0.86	3.18 ± 1.48	<0.001
Subcutaneous adipose tissue	4.87 ± 1.69	14.55 ± 4.6	<0.001

Data are presented as mean ± SD. P = P-values for comparison of unadjusted log_*e*_-transformed data by ANOVA.

### Study design

Intranasal insulin and placebo was administered on two separate study days (time-lag of 7–14 days) and fMRI was recorded. Studies were conducted after an overnight fast of at least 10 hours and started at 7.00 a.m. with a resting-state fMRI measurement under basal conditions (rsfMRI 1). After the basal measurement, an insulin/placebo spray was administered intranasally as described below. After 30 minutes, a second resting-state fMRI measurement was performed (rsfMRI 2).

Subjective feeling of hunger was rated at three time points (before spray application, 60 and 120 min after intranasal spray) on a visual analogue scale from 0 to 10 (0: not hungry at all; 10: very hungry) (Table 1). Venous blood samples were obtained at different time point (for details see Kullmann *et al*.^11^).

### Application intranasal insulin/placebo

The insulin and placebo spray were prepared in nasal sprays. In a randomized fashion, participants received on one day 160 U of insulin (Insulin Actrapid; Novo Nordisk, Bagsvaerd, Denmark) and on the other measurement day vehicle as placebo. Participants were single-blinded to the order of the conditions.

### Whole-brain fMRI Measurement

#### Data acquisition

Whole-brain fMRI data was obtained by using a 3.0 T scanner using a 12-channel head coil (Siemens Tim Trio, Erlangen, Germany). Functional data were collected by using gradient echo echo-planar imaging (GE-EPI) sequences. All participants were instructed not to focus their thoughts on anything in particular and to keep their eyes closed during the resting state fMRI acquisition. The following sequence was used: TR = 2 s, TE = 30 ms, FOV = 210 mm²,matrix 64 × 64, flip angle 90°, voxel size 3 × 3 × 3.6 mm^3^, slice thickness 3.6 mm, images were acquired in ascending order. Each brain volume comprised 26 axial slices and each functional run contained 176 image volumes, resulting in a total scan time of 6:04 minutes. In addition, high-resolution T1 weighted anatomical images (MPRage: 192 slices, matrix: 256 × 240, 1 × 1 × 1 mm^3^) of the brain were obtained.

#### Resting-state fMRI Data processing

We used the Data Processing Assistant for Resting-State fMRI (DPARSF)^67^ (http://www.restfmri.net) to analyze the resting state fMRI data. DPRSF is based on Statistical Parametric Mapping (SPM8) (http://www.fil.ion.ucl.ac.uk/spm) and Resting-State fMRI Data Analysis Toolkit^68^ (REST, http://www.restfmri.net). The functional images were realigned and co-registered to the T1 structural image. The anatomical image was normalized to the Montreal Neurological Institute (MNI) template using DARTEL, and the resulting parameter file was used to normalize the functional images (voxel size: 3 × 3 × 3 mm). Finally, the normalized images were smoothed with a three-dimensional isotropic Gaussian kernel (FWHM: 6 mm). A temporal filter (0.01~0.08 Hz) was applied to reduce low frequency drifts and high frequency physiological noise. Nuisance regression was performed using white matter, CSF, and the six head motion parameters as covariates. No participant had head motion with more than 2.0 mm maximum displacement or 2.0° of any angular motion.

#### Resting-state functional connectivity analyses

Functional connectivity (FC) maps were obtained using a seed-based voxel wise correlation approach by computing FC between a seed region and each voxel within the brain. We defined four seed regions according to Andrews-Hanna *et al*.^69^: the core region of the DMN and three prefrontal regions of DMN (seed 1: posterior cingulate/precuneus x: −8 y: −56 z: 26; seed 2: anterior medial prefrontal cortex x:−6 y: 52 z: −2; seed 3: dorsal medial prefrontal cortex x: 0 y: 52 z: 26 and seed 4: ventromedial prefrontal cortex x: 0 y: 26 z: −18 (Supplementary Figure 1). All seed regions included a 5 mm sphere. The FC maps were transferred to z values using Fisher’s transformation^68^. For further statistical analysis, FC maps of rs*fMRI2* were subtracted from rs*fMRI1* for both placebo and insulin day. Changes in FC 30 min after insulin versus placebo administration were analyzed in SPM8 using a full-factorial model (between group factor: lean and obese; within-group factor: insulin and placebo, covariate: oGTT-derived peripheral insulin sensitivity index). A statistical threshold of p_FWE_ < 0.05 voxel-level whole-brain corrected was applied. Additionally, small volume correction was performed for the hypothalamus and the hippocampus, as they are *a priori* regions of interest. The hypothalamus and hippocampus masks were based on the *wfu* pick atlas (http://fmri.wfubmc.edu/software/PickAtlas).

Functional connectivity values (z-transformed correlation coefficients) of regions showing significant change in FC after insulin administration were extracted for further statistical analyses in SPSS (version 20, IBM, Armonk, USA). We evaluated whether changes in FC show a significant relationship with the subjective feeling of hunger after insulin spray application, with peripheral insulin sensitivity, BMI and with different fat compartments as evaluated with T1-weighted whole-body MRI data (visceral adipose tissue (VAT), subcutaneous adipose tissue (SCAT) and total adipose tissue (TAT))^70^ using a multiple regression analyses. All regressions included age, total intracranial volume (TICV) and sex as confounding regressors. An adjusted statistical threshold of p ≤ 0.002 correcting for number of tests (n = 25) was used.

Mediation analyses of the relationship between VAT, hunger and extracted FC change between dorsal medial PFC and hippocampus was performed using PROCESS version 2.15 procedure in SPSS (www.afhayes.com). The concept of a mediation analysis works as follows. The relationship between a predictor (x) and an outcome variable (y) can be explained by their relationship to a third variable (the mediator (m)). The predictor (x) predicts the mediator (m) through the path denoted by path ***a***. The mediator (m) predicts the outcome (y) through the outcome denoted by path ***b***. The relationship between predictor and outcome controlling for the mediator in the model is denoted as direct effect i.e. path c’. The total effect is the effect of the predictor on the outcome when the mediator is not present in the model (i.e. path c). The effect of mediation is investigated through the indirect effect, which is a cross product of ***a*** and ***b*** ^71^. The mediation model included VAT as the predictor (**x**), change in hunger rating as the outcome variable (**y**) and change in FC between dorsal medial PFC and hippocampus as the mediator (**m**). Additional covariates were added to the model for adjustments (covariates: BMI, age, sex and TICV). The significance of the mediation analysis (i.e. indirect effect ***ab***) was estimated based on a bias-corrected bootstrap confidence interval (CI 95%, 10 000 bootstrap samples). If the confidence interval does not contain zero then the ‘true effect’ size is different from ‘no effect’. This means there is a significant mediation. Furthermore, we report a standardized indirect effect measure to ensure comparability with other studies^71^.

## Electronic supplementary material

Supplementary Figures
