# Supplementary material for: Intranasal insulin enhances brain functional connectivity mediating the relationship between adiposity and subjective feeling of hunger
Source: Sci Rep. 2017 May 9;7:1627. doi: 10.1038/s41598-017-01907-w (PMC5431641; doi:10.1038/s41598-017-01907-w)
Supplement: Supplementary file 1 — Supplementary Figures [file 41598_2017_1907_MOESM1_ESM.pdf]

Supplementary file

Intranasal insulin enhances brain functional connectivity mediating the relationship between adiposity and subjective feeling of hunger

Stephanie Kullmann[1,2,3], *Martin Heni*[1,2,4], *Ralf Veit*[1,2,3], *Klaus Scheffler*[5,6], *Jürgen Machann* [1,2], *Hans-Ulrich Häring* [1,2,4], *Andreas Fritsche* [1,2,4], *Hubert Preissl* [1,2,7,8]

1) Institute for Diabetes Research and Metabolic Diseases of the Helmholtz Center Munich at the University of Tübingen, Tübingen, Germany
2) German Center for Diabetes Research (DZD e.V.), Tübingen, Germany
3) Institute of Medical Psychology and Behavioral Neurobiology, University of Tübingen, Germany
4) Department of Internal Medicine IV, University of Tübingen, Germany
5) Department of High-Field Magnetic Resonance, Max Planck Institute for Biological Cybernetics, Tübingen, Germany
6) Department of Biomedical Magnetic Resonance, University of Tübingen, Germany
7) Institute for Diabetes and Obesity, Helmholtz Diabetes Center at Helmholtz Zentrum München, German Research Center for Environmental Health (GmbH), Neuherberg, Germany
8) Institute of Pharmaceutical Sciences, Department of Pharmacy and Biochemistry, Eberhard Karls Universität Tübingen, Tübingen, Germany

***Corresponding author:**

Dr. Stephanie Kullmann

fMEG Center

University of Tübingen

Otfried Müller Strasse 47

72076 Tübingen, Germany

Phone: ++49-(0)7071-2987703

Fax: ++49-(0)7071-295706

Email: stephanie.kullmann@med.uni-tuebingen.de

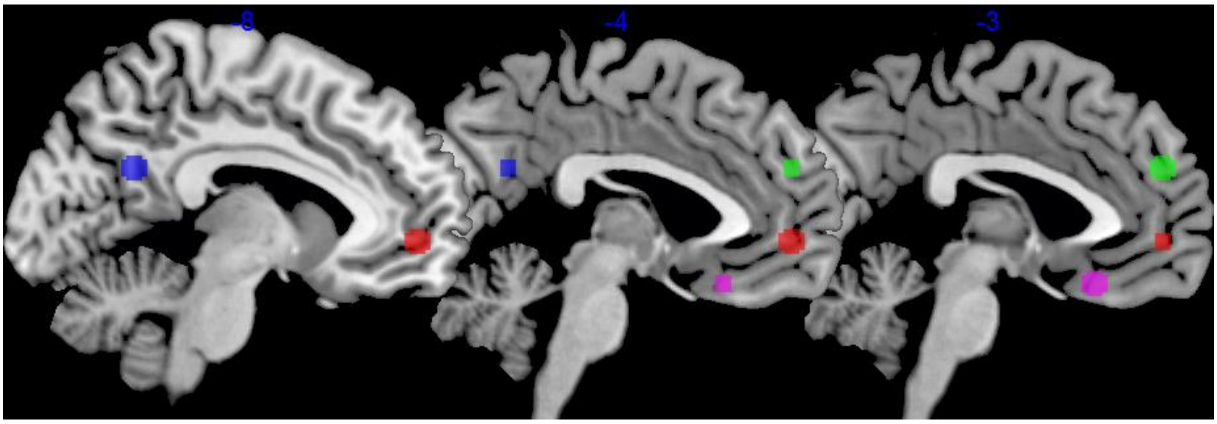

Supplementary Figure 1. Default-mode network (DMN) seed regions based on Hanna-Andrews et al., displayed on a standard anatomical image (sagittal view). The four seed regions are indicated by different colors. Blue: posterior cingulate/precuneus x: -8 y: -56 z: 26 (seed 1); Red: anterior medial prefrontal cortex x:-6 y: 52 z: -2 (seed 2); Green: dorsal medial prefrontal cortex x: 0 y: 52 z: 26 (seed 3); Violet: ventromedial prefrontal cortex x: 0 y: 26 z: -18 (seed 4). All seed regions included a 5 mm sphere. Functional connectivity maps of the DMN were obtained using a seed-based voxel wise correlation approach by computing functional connectivity between the seed region and each voxel within the brain.

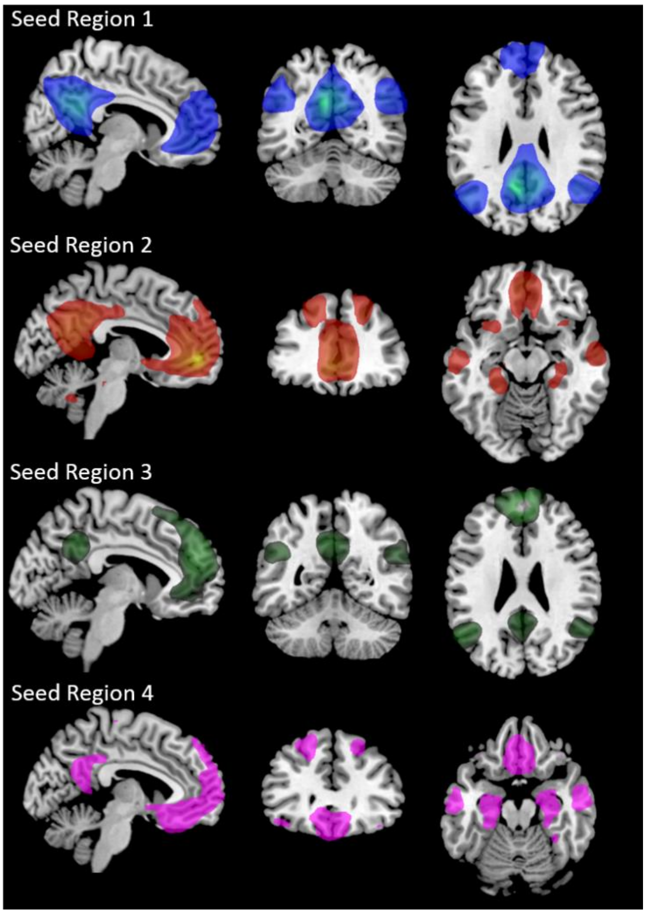

Supplementary figure 2. Group-averaged default mode network (DMN), based on Hanna-Andrews et al., of lean and overweight/obese participants under baseline condition (rs*fMRI1*) for each seed separately (p_{FWE}<0.05, whole-brain corrected). Functional connectivity maps of the DMN were obtained using a seed-based voxel wise correlation approach by computing functional connectivity between the seed region and each voxel within the brain. The four seed regions are indicated by different colors. Blue: posterior cingulate/precuneus x: -8 y: -56 z: 26 (seed 1); Red: anterior medial prefrontal cortex x:-6 y: 52 z: -2 (seed 2); Green: dorsal medial prefrontal cortex x: 0 y: 52 z: 26 (seed 3); Violet: ventromedial prefrontal cortex x: 0 y: 26 z: -18 (seed 4). All seed regions included a 5 mm sphere.
